# The *in vitro* direct mycobacterial growth inhibition assay (MGIA) for the early evaluation of TB vaccine candidates and assessment of protective immunity: a protocol for non-human primate cells

**DOI:** 10.12688/f1000research.51640.1

**Published:** 2021-03-30

**Authors:** Rachel Tanner, Emily Hoogkamer, Julia Bitencourt, Andrew White, Charelle Boot, Claudia C. Sombroek, Stephanie A. Harris, Matthew K. O'Shea, Daniel Wright, Rachel Wittenberg, Charlotte Sarfas, Iman Satti, Frank A.W. Verreck, Sally A. Sharpe, Helen A. Fletcher, Helen McShane

**Affiliations:** 1 Nuffield Department of Medicine, The Jenner Institute, Oxford, OX3 7DQ, UK; 2 Public Health England, Salisbury, SP4 0JG, UK; 3 Gonҫalo Moniz Institute, Oswaldo Cruz Foundation (FIOCRUZ), Salvador, 40296-710, Brazil; 4 Department of Parasitology, Biomedical Primate Research Centre, Rijswijk, 2288 GJ, The Netherlands; 5 Institute of Immunology and Immunotherapy, University of Birmingham, UK, Birmingham, B15 2TH, UK; 6 London School of Hygiene and Tropical Medicine, London, WC1E 7HT, UK

**Keywords:** 3Rs, refinement, non-human primate, macaque, mycobacterial growth inhibition assay, tuberculosis, vaccines

## Abstract

The only currently available approach to early efficacy testing of tuberculosis (TB) vaccine candidates is *in vivo *preclinical challenge models. These typically include mice, guinea pigs and non-human primates (NHPs), which must be exposed to virulent *M.tb* in a ‘challenge’ experiment following vaccination in order to evaluate protective efficacy. This procedure results in disease development and is classified as ‘Moderate’ in severity under EU legislation and UK ASPA licensure. Furthermore, experiments are relatively long and animals must be maintained in high containment level facilities, making them relatively costly. We describe an *in vitro* protocol for the direct mycobacterial growth inhibition assay (MGIA) for use in the macaque model of TB vaccine development with the aim of overcoming some of these limitations. Importantly, using an *in vitro* assay in place of *in vivo M.tb* challenge represents a significant refinement to the existing procedure for early vaccine efficacy testing. Peripheral blood mononuclear cell and autologous serum samples collected from vaccinated and unvaccinated control animals are co-cultured with mycobacteria in a 48-well plate format for 96 hours. Adherent monocytes are then lysed to release intracellular mycobacteria which is quantified using the BACTEC MGIT system and colony-forming units determined relative to an inoculum control and stock standard curve. We discuss related optimisation and characterisation experiments, and review evidence that the direct NHP MGIA provides a biologically relevant model of vaccine-induced protection. The potential end-users of the NHP MGIA are academic and industry organisations that conduct the assessment of TB vaccine candidates and associated protective immunity using the NHP model. This approach aims to provide a method for high-throughput down-selection of vaccine candidates going forward to *in vivo* efficacy testing, thus expediting the development of a more efficacious TB vaccine and offering potential refinement and reduction to the use of NHPs for this purpose.

Research highlights **Scientific benefits**

- Potential to expedite the development of a much-needed effective TB vaccine through rapid down-selection of candidates at an early stage.
- Tractable system for the exploration of immune mechanisms underlying the control of mycobacterial growth.
- Opportunity to biologically validate the direct PBMC MGIA through correlation with protection from *in vivo M.tb* challenge on an individual animal basis.

**3Rs benefits**

- Refining early efficacy testing of TB vaccine candidates by using the MGIA in place of *in vivo* infection with pathogenic *M.tb*.
- Reducing the number of NHPs used in TB vaccine testing and associated immunology studies by down-selecting the number of candidates going forward to *in vivo* testing and by allowing the testing of multiple conditions using cells from a single group.
- Bridging of the assay to use in target species including humans to replace the use of preclinical models in some settings.

**Practical benefits**

- Measures of vaccine efficacy obtained more rapidly than *in vivo M.tb* challenge studies (2 weeks vs. 12 weeks routinely required for *in vivo* challenge). Quantification using the BACTEC MGIT system also more rapid than conventional colony counting on agar.
- Negates the need for high containment animal facilities required for *in vivo M.tb* challenge.
- More cost-effective, much lower resource requirement and less technically challenging than *in vivo M.tb* challenge studies in NHPs.

**Current applications**

- Assessing the BCG vaccine-induced response as a benchmark and comparing between different routes of administration ^37^.
- Comparing outcomes with levels of protection from *in vivo M.tb* or BCG challenge to determine biological validity ^37^.
- Applying to other aspects of TB research, such as assessing ability to control mycobacterial growth following *M.tb* infection and comparisons between species ^36^.
- Exploring underlying immune mechanisms including associations between growth inhibition and various cell type frequencies, specific antibodies, and baseline characteristics ^35^, [Tanner R, unpublished data].

**Potential applications**

- Assessing protective efficacy of novel TB vaccine candidates.
- Understanding associated immune mechanisms of protection.
- Measuring vaccine potency, lot-to-lot consistency and stability.
- Adaptation for use with other pathogens (e.g. *S. aureus*).

## 1.0 Introduction

Approximately 1 in 4 people globally are infected with tuberculosis (TB), with 10 million new infections and 1.4 million deaths reported in 2019 ^1^. This serious public health threat is further exacerbated by the spread of multi- and extensively-drug resistant strains of the causative agent, *Mycobacterium tuberculosis* ( *M.tb*) ^2^. An efficacious vaccine is widely acknowledged to be the most effective intervention strategy. The Bacillus Calmette-Guérin (BCG) vaccine, first introduced in 1921, remains the only currently-licenced TB vaccine. Although protective in infants against severe forms of TB disease, BCG affords extremely variable levels of protection against the most common and infectious form of TB, pulmonary disease, in adults ^3^. BCG-induced protection against pulmonary TB is lowest in regions close to the equator such as sub-Saharan Africa and India where an effective vaccine is most desperately needed ^3^. However, development of a successful TB vaccine is severely hampered by the lack of a validated correlate or biomarker of protection ^4^. It remains unclear which aspects of the immune response confer protection from TB disease, and therefore which parameters to target with a vaccine and to assess as a reliable measure of protective efficacy.

### 1.1 Existing approaches to early evaluation of TB vaccine efficacy

In the absence of a validated immune correlate of protection from TB, the only currently available approach to early efficacy testing of TB vaccine candidates is the use of preclinical ‘challenge’ (infection) models. Animals used typically include mice, guinea pigs and non-human primates (NHPs). *In vivo* testing offers the obvious and unparalleled advantage of modelling the complexities of biological systems (the immune system representing one of the most complex and systemic of all) in the context of their natural microenvironment over time. NHPs are considered the most representative model for human TB due to their anatomical and physiological similarities, natural susceptibility to *M.tb* infection and comparable pathological and clinical outcomes ^5^. Rhesus and cynomolgus macaques in particular are widely used in TB vaccine studies as BCG vaccination offers partial and quantifiable protection against *M.tb* challenge in these species ^6– 10^. There has been recent emphasis on the use of NHPs as the ‘gatekeeper’ for progression of TB vaccine candidates to clinical trials, and the numbers used in the field are increasing ^11^.

In order to evaluate the protective efficacy of a candidate TB vaccine, animals must be exposed to virulent *M.tb* in a ‘challenge’ experiment following vaccination. Infection with *M.tb* results in disease development and is classified as ‘Moderate’ in severity under EU legislation and UK ASPA licensure ^12^. Welfare considerations include the infection process itself, disease symptoms, and the definition of humane endpoints. A study assessing the lifetime experience of macaques found that the combined welfare assessment score increased from <10 to >50 following *M.tb* challenge, reflecting a decline in procedural, physical, psychological and environmental welfare ^13^. Other limitations of the NHP challenge model in TB vaccine testing include the long and costly nature of such experiments, and have been discussed further elsewhere ^14^.

One potential alternative or complementary tool for assessing vaccines is functional *in vitro* assays such as growth inhibition assays (GIAs) as a potential surrogate measure of vaccine efficacy. Such assays aim to provide unbiased read-outs of the combined effects of the host immune response, strain virulence and influences of interventions. They have been applied with some degree of success to a range of other disease models including HIV, malaria and meningitis ^15– 17^. A number of mycobacterial GIAs (MGIAs) for TB have been previously described in the literature, including the use of reporter strains in whole blood ^18, 19^ and primary or secondary lymphocyte/monocyte co-cultures in humans ^20, 21^, bone marrow macrophage/splenocyte cultures in mice ^22, 23^, and cattle peripheral blood mononuclear cell (PBMC) ^24, 25^. These have been comprehensively reviewed elsewhere ^26^. However, in all cases such assays are technically challenging and limited follow-up work has been conducted to qualify an MGIA that could be transferred across laboratories using a standardised, reproducible method.

We have previously worked to optimise and standardise a simplified MGIA (known as the ‘direct MGIA’) for use in humans and mice, adapted from methods originally described by Wallis *et al*., using the BACTEC MGIT mycobacterial quantification system ^27– 29^. Applying this approach, we have demonstrated a BCG vaccine-induced effect in these species ^30– 32^, and an association with *in vivo* protection from mycobacterial challenge has also been described ^33, 34^. Importantly, preclinical MGIAs represent a potential alternative to the *in vivo M.tb* challenge step in early TB vaccine testing: a major refinement which is particularly important for NHPs due to the additional welfare and behavioural considerations that apply when using these species in medical research. Furthermore, the NHP model represents a unique opportunity for biological validation of the assay against direct measures of *in vivo* protection, as discussed below, permitting bridging to use in other species including humans. We present a protocol for the first example of an NHP MGIA using *in vitro* cell co-culture, adapted from our direct MGIA methods described in humans and mice, with the aim of refining and expediting early TB vaccine testing ^35– 37^.

### 1.2 3Rs relevance

***1.2.1 Refinement***. The main 3Rs objective of the direct NHP MGIA is to provide a potential refinement to the process of early TB vaccine testing in NHP models through offering a functional *in vitro* assay as an alternative to *in vivo* infection with pathogenic *M.tb*. If the MGIA were applied in place of *in vivo M.tb* infection, animals would still be required for vaccination, but blood samples could be taken before and at various time-points after vaccination, and ability to control growth of mycobacteria assessed *in vitro* without the need for *M.tb* infection of the animals. Lifetime experience would be improved and the severity rating for the experiment would be downgraded from ‘Moderate’ to ‘Mild’ under EU legislation ^12, 13, 38^.

***1.2.2 Reduction***. A successful, validated MGIA could be used to test and down-select experimental TB vaccine candidates at an early stage of development, reducing the number going forward to virulent *M.tb* challenge experiments and therefore the number of animals used. It would also reduce the number of animals required as multiple conditions (such as control of different mycobacterial strains or contribution of different immune parameters) could be tested using samples from a single group of vaccinated animals. It is increasingly acknowledged that the level of protection conferred by a TB vaccine candidate may be influenced by the *M.tb* strain/s prevalent in the geographical region in which it is being tested ^39, 40^. Furthermore, preclinical testing of TB vaccine candidates generally use standardised (and sometimes attenuated) laboratory strains of *M.tb* as challenge agents. It is prudent to test vaccine efficacy against a range of *M.tb* strains or clinical isolates to ensure widespread applicability. Using an *in vivo* challenge model, this would necessitate an additional group of experimentally infected animals for each strain. The NHP MGIA allows samples from a single group of animals to be assessed for ability to control multiple mycobacterial strains, particularly as there are no restrictions on the inoculum, unlike for assays which use reporter strains for example.

The MGIA also offers a tractable model for the exploration of underlying immune mechanisms involved in the control of mycobacterial growth. Cell types of interest may be depleted, purified and added back at different concentrations, pathways interrupted, receptors blocked and so forth to elucidate those of importance. The ability to conduct such experiments *in vivo*, for example adoptive transfer, is limited and requires large numbers of animals. Using the MGIA, multiple conditions can be explored with a sample set from a single group of animals, in contrast to the additional groups required for equivalent *in vivo* experiments with the associated impacts on disease severity. To illustrate, a hypothetical *in vivo* experiment designed to test a novel vaccine candidate would require minimum group sizes of 8 macaques to detect a nine-AU reduction in total pathology given a group standard deviation of 5.8 with a power of 80% and an α of 0.05. Including a naïve control group, a BCG-vaccinated group as a benchmark, and a group for the novel vaccine candidate, testing efficacy against three strains of *M.tb* would require three groups for each condition (as each animal can only be challenged with one strain) = 72 animals vs. 24 for *in vitro* assessment using the NHP MGIA ( Table 1). This represents a 3-fold reduction in the number of animals used.

**Table 1.** A hypothetical experimental design demonstrating the numbers of animals required for *in vivo* challenge vs. *in vitro* MGIA evaluation.

Method	No. of animals per group	No. for vaccine candidate	No. for *M.tb* strains	Total no. of animals
*In vivo* challenge	8 8 8	1 x naïve 1 x BCG 1 x vaccine X	x 3 strains = 24 x 3 strains = 24 x 3 strains = 24	72
*In vitro* MGIA	8 8 8	1 x naïve 1 x BCG 1 x vaccine X	x 3 strains = 8 x 3 strains = 8 x 3 strains = 8	24

**Figure 1.**
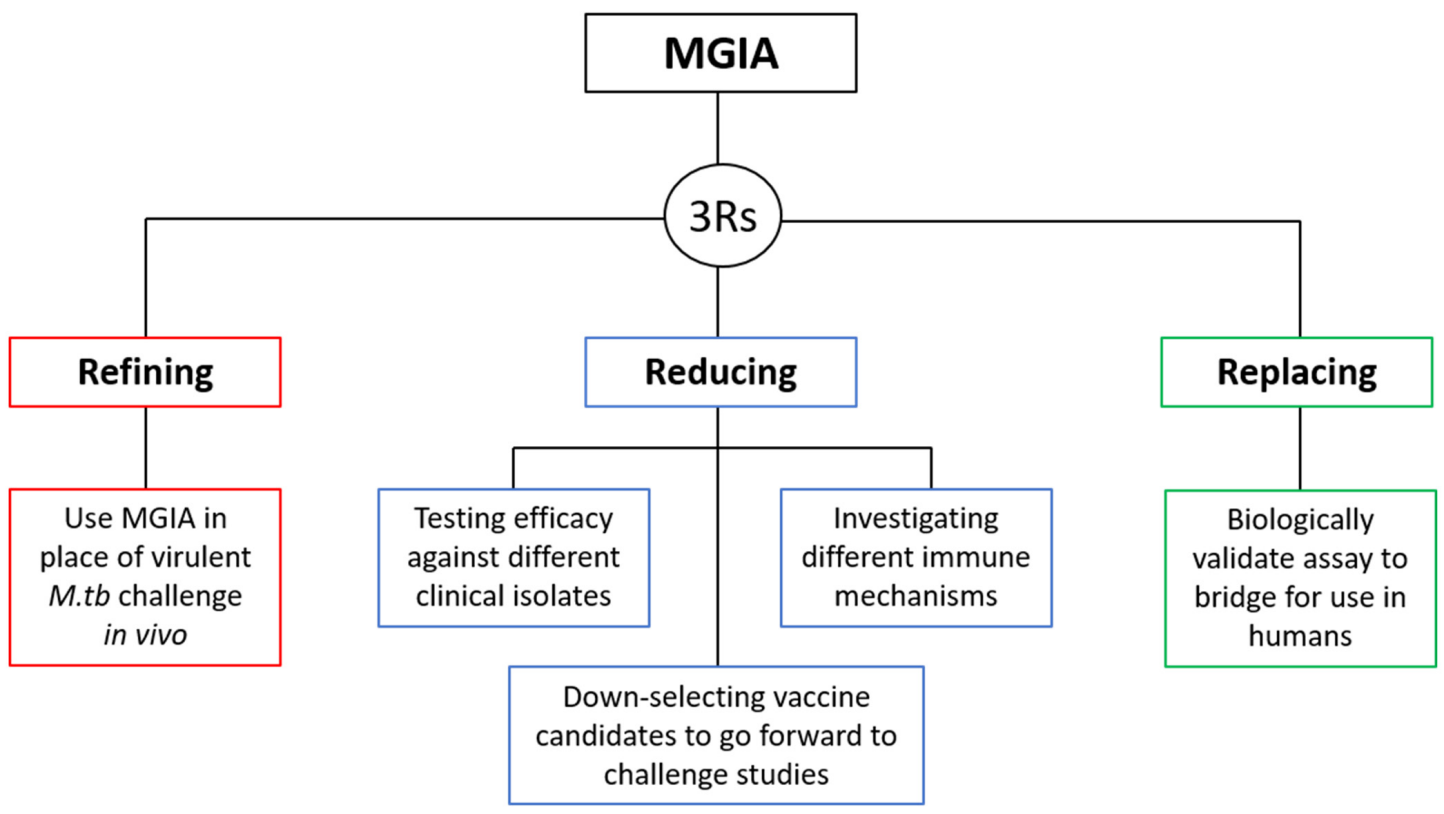
The 3Rs relevance of the NHP MGIA.

***1.2.3 Replacement***. As described, our direct MGIA method has also been optimised for use with human cells ^29^. However, the biological relevance of the direct MGIA, as for any potential correlate of protection, can only be confirmed by demonstrating an association with *in vivo* protection from either controlled or natural infection or disease. While controlled infection with BCG may be used as a potential surrogate in human challenge studies, virulent *M.tb* cannot ethically be used ^33^. The NHP model provides an opportunity to validate the assay against protection from *M.tb* as well as BCG infection, allowing greater confidence in the relevance of the human assay such that preclinical models such as mice, guinea pigs and NHP may ultimately be replaced with human samples in some settings. Furthermore, the MGIA could be applied in the measurement of vaccine potency, lot-to-lot consistency and stability as an alternative to *in vivo* infection experiments. The 3Rs relevance of the NHP MGIA is summarised in Figure 1.

### 1.3 Potential end-users

The potential end-users of the NHP MGIA are academic and industry organisations that conduct the assessment of TB vaccine candidates and associated protective immunity using the NHP model. This currently includes groups in Europe such as the UK, Sweden and the Netherlands; Pittsburgh, Chicago, Boston, California, New Orleans, Texas and Seattle in the USA; Osaka, Japan; Wuhan, China; and the Philippines among others ^41^. Peña *et al.* reviewed the major NHP TB studies published between 2001 and 2014. During this period, the mean number of publications increased from 1 per year in 2001–2007 to 4 per year in 2007–2014. The mean number of animals used per publication was 14 for rhesus macaques (range 3–32) and 20 for cynomolgus macaques (range 2–44). For TB vaccine-related studies specifically, the mean number of animals used was 20 (range 12–32) ^41^. Conducting a literature search for the time-period 2019–2020 using the Google Scholar search terms ‘tuberculosis’ and ‘macaque’, and excluding results relating to TB diagnostics or drugs, and/or SIV coinfection, we identified 21 publications reporting NHP studies of TB vaccines and/or TB immunology employing a mean of 27 animals (range 6–75), the majority of which involved challenge with *M.tb*. This illustrates a trend towards increased use of NHPs in the field and larger group sizes. Widespread adoption of a validated NHP MGIA could significantly reduce the number of animals undergoing infection with virulent *M.tb* and potentially the overall numbers used. 

## 2.0 Methods

### 2.1 Materials

The reagents and equipment required for the direct NHP MGIA are described in Table 2 and Table 3 respectively. It is not essential to use a specific supplier of reagents or equipment unless it is specified in the table.

**Table 2.** Reagents required for the direct PBMC NHP MGIA.

Reagents
RPMI-1610 (serum-free)
RPMI-1640 medium with HEPES modification
L-glutamine (200mM 100X)
Sodium Pyruvate
Foetal Bovine Serum (FCS)
Benzonase nuclease (25U/µl)
BBL MGIT tubes containing 7ml media (Becton Dickinson)
PANTA/enrichment supplement for MGIT tubes (Becton Dickinson)
Cell culture grade sterile water
Standardised BCG Pasteur stock (Aeras)
Phosphate Buffered Saline (PBS)
Tween 80
Middlebrook 7H10 Agar base
Oleic Albumin Dextrose Catalase (OADC) supplement
Glycerol

**Table 3.** Equipment required for the direct PBMC NHP MGIA.

Equipment
BACTEC MGIT 320/960 instrument (Becton Dickinson)
37°C water bath
Centrifuge and microcentrifuge
37°C incubator with CO _2_
Cell counter/microscope and associated equipment
48-well tissue culture plates
2ml screw-cap tubes
Vortex
360° tube rotator
Parafilm
Sterile borosilicate solid-glass beads (1mm)
50ml falcon tubes
Petri dishes (60mm)
P20, P200 and P1000 pipettes and filter tips

### 2.2 Samples

***2.2.1 NHP samples***. Stored samples used in the optimisation experiment shown in Figure 6 were collected from n=7 female rhesus macaques of Indian genotype aged 14–15 years as part of a study of BCG vaccination conducted at Public Health England (PHE) in the UK. Design and procedures of the original study were approved by the Public Health England Animal Welfare and Ethical Review Body and authorized under an appropriate UK Home Office project license. Animals were housed in compatible social groups in accordance with the Home Office (UK) Code of Practice for the Housing and Care of Animals Used in Scientific Procedures (1989) and the National Centre for Refinement, Reduction and Replacement (NC3Rs) Guidelines on Primate Accommodation, Care and Use, August 2006 (NC3Rs, 2006). They were provided with enrichment in the form of food and non-food items on a daily basis; animal welfare was monitored daily. Animals were captive-bred for research purposes, were obtained from established breeding colonies at PHE, were healthy and had not been used previously for experimental procedures. Animals were sedated by intramuscular (IM) injection of ketamine hydrochloride (Ketaset, 100 mg/ml, Fort Dodge Animal Health Ltd, Southampton, UK; 10 mg/kg) for procedures requiring removal from their housing. Animals were weighed, had rectal temperature measured and were examined for gross abnormalities whenever procedures (vaccination, blood sample collection) were conducted. There were no adverse events, and no humane endpoints for this study as it did not involve *M.tb* challenge.

***2.2.2 Human samples***. Samples used in the optimisation experiments shown in Figure 5 and Figure 7 were obtained from volunteers at the Jenner Institute, Oxford, in accordance with University of Oxford policy. All human samples were collected in accordance with the ethical principles set forth in the Declaration of Helsinki as agreed by the World Medical Association General Assembly (Washington 2002), ICH Good Clinical Practice (GCP) and local regulatory requirements; volunteers gave written informed consent.

### 2.3 Design of optimisation experiments

For the experiments shown in Figure 5, the experimental unit was a co-culture containing 1 × 10 ^6^ cells from a single volunteer to ensure all variables were constant apart from the one under investigation (treatment of mycobacterial stock). The sample sizes were duplicate co-cultures for Figure 5A as this was a time-course with repeated measures of a single condition, and (n=6) replicate co-cultures for Figure 5B as this was a group-wise comparison of different stock conditions. For the experiments shown in Figure 6 and Figure 7, the experimental unit was an individual macaque (n=7) or an individual volunteer (n=6) respectively each tested in duplicate; these were group-wise comparisons of co-cultures containing different serum/plasma conditions. Samples were not selected but were used according to availability and recovery of a sufficient number of PBMC post-thawing and a sufficient volume of serum/plasma. No data points were excluded from the analysis. Minimum sample size (n=6) for these experiments was calculated based on the effect size of 0.2 log _10_ CFU (colony-forming units) observed in previous MGIA experiments considered to be biologically relevant (given matched measures of *in vivo* efficacy) and estimates of variability within a group with a power of 80% and an α of 0.05. Cells were allocated to conditions by pipetting to mix and adding to conditions in repeated sequence where relevant ( Figure 5B). Operator blinding was not possible because the comparisons required operator interventions in the laboratory and the BCG status of the animals was not relevant to these experiments. In all cases, the outcome measured was effect of co-culture condition (stock treatment, plasma vs. serum, or collection/treatment of serum) on mycobacterial growth over the 96 hour co-culture period, as measured by MGIT time-to-positivity (TTP) and/or converted to log _10_ CFU normalised to the direct-to-MGIT inoculum control. Statistical analysis was conducted using GraphPad Prism v.7, and data was analysed using non-parametric tests due to the small sample sizes; multi-group data was corrected for multiple comparisons using Dunn’s test (all conditions vs. all other conditions). Following confirmation of normality in the distribution of differences between paired measurements, the Bland-Altman method was used to compare MGIA outcomes between serum and plasma in Figure 6. 95% confidence intervals for the Bland-Altman limits of agreement were calculated using the methods described by Carkeet ^42^.

### 2.4 Mycobacterial Growth Inhibition Assay

***2.4.1 PBMC preparation***. Cryopreserved PBMC were rapidly thawed in a water bath at 37°C until a small amount of frozen material remained. Samples were gradually added to 10ml RPMI (containing 10% foetal calf serum and 2mM L-glutamine) using a Pasteur pipette. The cryovial was rinsed using 1ml of fresh medium and added to the corresponding tube, which was then centrifuged at 350 *g* for 7 min. Supernatants were removed by inversion and cells resuspended at an approximate concentration of 2 × 10 ^6^ cells per ml of RPMI (containing 10% foetal calf serum and 2mM L-glutamine) and 2µl/ml of 25 U benzonase added to each tube. Cells were rested at 37°C for 2 h with 5% CO _2_ before counting using an automated CASY cell counter.

***2.4.2 MGIA***. For the human MGIA experiments shown in Figure 5 and Figure 7, 600µl RPMI (containing 2mM L-glutamine and 25mM HEPES) seeded with 1 × 10 ^6^ PBMC and ~100 CFU ( Figure 5) or ~500 CFU ( Figure 7) BCG Pasteur was added to duplicate 2ml screw-cap tubes. The co-cultures were incubated on a 360° rotator at 37°C for 96 hours, after which time tubes were microcentrifuged at 15,300 *g* for 10 minutes and the supernatant carefully removed by pipetting. Cells were lysed with the addition of 500µl sterile water and the tubes pulse-vortexed at 0, 5 and 10 minutes. For the NHP direct PBMC ‘in-plate’ MGIA, shown in Figure 6, 3 × 10 ^6^ PBMC and ~500 CFU BCG Pasteur in a total volume of 480μl RPMI (containing 2mM L-glutamine and 25mM HEPES), plus 120μl autologous serum or plasma matched to animal were added per well of a 48-well plate (total volume 600μl per well). Co-cultures were incubated at 37°C for 96 hours with CO _2_ and then transferred to 2ml screw-cap tubes and centrifuged at 15,300 *g* for 10 minutes. During this time, 500μl sterile water was added to each well to lyse adherent monocytes and release intracellular mycobacteria. Supernatants were carefully removed from the 2ml screw-cap tubes by pipetting, and water from the corresponding well added to the remaining pellet. In all cases, tubes were pulse vortexed and lysates transferred to a BACTEC MGIT tube supplemented with PANTA antibiotics (polymyxin B, amphotericin B, nalidixic acid, trimethoprim and azlocillin) and OADC enrichment broth (Becton Dickinson, UK) before being placed on the BACTEC 960 machine (Becton Dickinson, UK) and incubated at 37°C until the detection of positivity by fluorescence. On day 0, duplicate direct-to-MGIT control tubes were set up by inoculating supplemented BACTEC MGIT tubes with the same amount of mycobacteria as the samples. The TTP read-out can be converted to log _10_ CFU using stock standard curves of TTP against inoculum volume and CFU. ‘Normalised mycobacterial growth’ is equal to (log _10_ CFU of sample – log _10_ CFU of growth control).

## 3.0 Detailed protocol for the NHP direct PBMC MGIA

*Note: All work must be performed under sterile tissue culture conditions in a Class II biological safety cabinet and filter tips should be used throughout.*

### 3.1 At least three weeks ahead of time, generate a standard curve as follows (summarised graphically in Figure 2)

**Figure 2.**
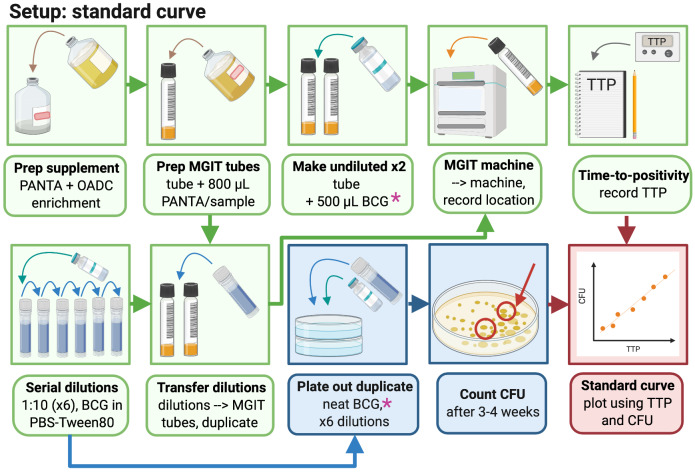
Generation of a stock standard curve (created with BioRender.com).

3.1.1 Thaw one vial of BCG Pasteur (or other desired mycobacterial strain) at room temperature.

*Note: We recommend a standardised stock of BCG Pasteur produced by Aeras specifically for use in the direct MGIA for consistency. Other stocks may be used but clumping may compromise assay reproducibility and sensitivity; if this is the case, vortexing with ~50 × 1mm borosilicate glass beads for 2 minutes prior to inoculation is recommended to reduce clumping.*

*Note: If M.tb strains are used, all work should be conducted in an appropriate high biosafety containment laboratory.*

3.1.2 Prepare 6 sterile 2ml screw-cap tubes by adding 1.35ml sterile PBS to each tube and labelling 1 to 6.

3.1.3 Add 150µl neat BCG Pasteur stock to tube 1, mix by pipetting up and down, and then take 150µl from this tube and add to tube 2, and so on to make a 1:10 dilution series.

*Note: The contents of each tube should be mixed thoroughly by pipetting up and down several times before adding to the next tube in the dilution series.*

3.1.4 Prepare MGIT supplement medium by pouring one bottle of OADC growth enrichment into one bottle of lyophilised PANTA. Mix by inverting several times until fully dissolved.

*Note: Enrichment media should be used on day of reconstitution.*

3.1.5 Add 800µl of supplement medium to each of 14 BACTEC MGIT tubes (2 per standard curve dilution).

*Note: Tubes are oxygen-enriched and time without caps should be minimised.*

*Note: MGIT tubes should be used on day of supplementation and should not be stored following supplementation.*

3.1.6 Add 500µl of neat BCG stock directly to each of two supplemented MGIT tubes and invert to mix.

3.1.7 Add 500µl from each of dilution tubes 1–6 to MGIT tubes in duplicate and invert to mix.

*Note: After addition of BCG, MGIT tubes should be capped immediately and inverted to mix.*

3.1.8 Scan MGIT tube barcodes on the BACTEC MGIT machine and place in the indicated slots.

*Note: The machine will generate an alarm when tubes reach a predefined level of fluorescence (indicating that mycobacteria have utilised the oxygen previously quenched to the fluorochrome). Positive tubes will be indicated by flashing lights and can be scanned out of the machine and the corresponding TTP recorded.*

3.1.9 Divide four 7H10 agar plates into quadrants and spot 3 × 20µl from the neat vial and each dilution into a quadrant on each of two plates. Leave plates to dry in the Class II cabinet before sealing with parafilm and placing in a CO _2_ incubator at 37°C. Plates should be checked after 2 weeks and daily henceforth; as soon as colonies are visible they should be counted and the number of spots recorded for each dilution and averaged across the 3 replicates.

3.1.10 Generate a standard curve by plotting TTP against CFU for each input volume and use regression analysis to obtain an equation for the curve. CFU should be fitted using a semi-log line, and log _10_ CFU with a linear regression. Solve the equation describing the line for X: Y = A*X+B -> X = (Y-B)/A where A = slope and B = y-intercept). By inserting the TTP (=Y) for any given sample, the corresponding number of CFU (or log _10_ CFU) can now be calculated. Further information including a sample MGIT read-out and standard curve have previously been provided by Zelmer *et al.* ^43^.

### 3.2 MGIA day 0: Assay set-up (summarised graphically in Figure 3)

**Figure 3.**
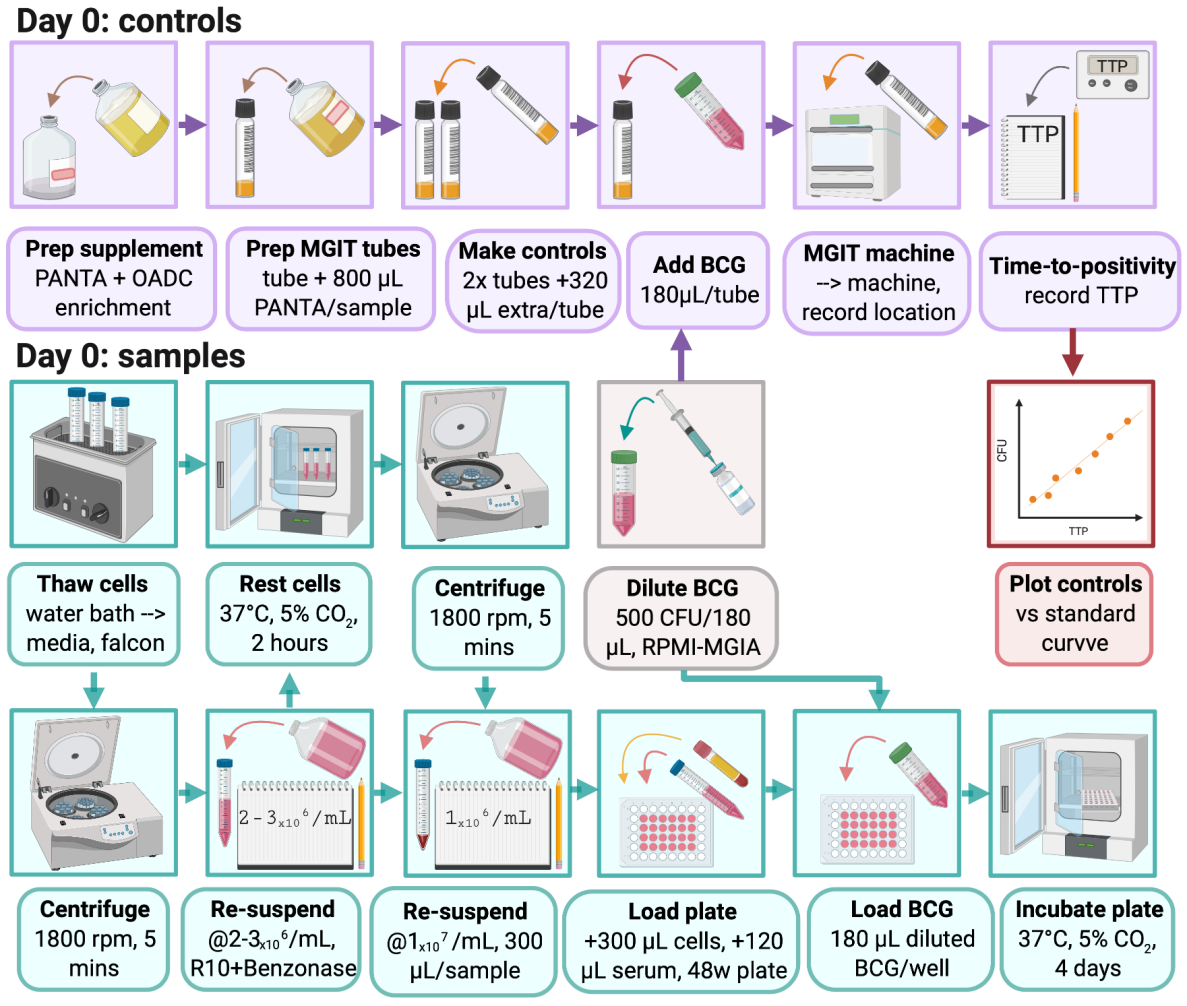
Day 0 MGIA set-up (created with BioRender.com).

3.2.1 Thaw cryopreserved cells by holding the lower portion of the vial in a 37°C water bath.

*Note: Vials should be removed from the water bath when a small amount of frozen material is still visible and the outside of the vial should be cleaned with 70% ethanol.*

3.2.2 Pipette cells up and down gently using a Pasteur pipette and gradually add to 10ml RPMI (with L-glutamine and sodium pyruvate but NO antibiotics (pen/strep)).

*Note: Prepare labelled tubes with 10ml media before beginning the thawing process.*

3.2.3 Rinse out the cryovial contents with 1ml of fresh medium and add the remaining cells.

3.2.4 Centrifuge at 350 *g* for 7 minutes.

3.2.5 Pour off supernatant and resuspend cells at approximately 2 – 3 × 10 ^6^ cells per ml of RPMI (with L-glutamine and sodium pyruvate but no antibiotics (pen/strep)).

3.2.6 Rest for 2 hours with loosened caps in a 37°C incubator with 5% CO _2_.

3.2.7 Count viable cells using standard methods (such as a haemocytometer or automated cell counter) and resuspend at 10 × 10 ^6^ cells per ml of RPMI (with 2mM L-glutamine and 25mM HEPES but NO antibiotics (pen/strep)).

3.2.8 Place 300μl of cell mix (containing 3×10 ^6^ PBMC) into labelled wells of a 48-well plate.

*Note: Replicate cultures should be performed if sufficient cells are available, but replicates have been demonstrated to be consistent (coefficient of variation (CV) <10%) ^37^ such that a single culture is acceptable where cell availability is limiting.*

*Note: Do not use wells on the outside rows/columns of the 48-well plate for cultures. These should contain 600μl of RPMI medium only.*

3.2.9 Add 120µl of non-heat inactivated autologous serum or plasma matched to the animal and time-point (to give a final concentration of 20%).

*Note: Serum should be kept sterile or syringed through a 0.2µM cellulose acetate filter prior to use.*

*Note: 10% filtered pooled human AB serum may be used if autologous serum is not available, but will not capture the influence of serum factors such as antibodies on control of mycobacterial growth.*

*Note: Autologous plasma may be used if serum in unavailable (see section 4.1.2.3); if plasma is viscous, it may be warmed in a 37°C incubator.*

*Note: Ensure serum/plasma is mixed well (for example by briefly vortexing) before adding.*

3.2.10 Thaw BCG stock at room temperature and prepare to the correct concentration in RPMI (with 2mM L-glutamine and 25mM HEPES but NO PEN/STREP). The appropriate dilution factor will depend on the particular stock, but should be calculated using the standard curve generated in section 3.1 to give a concentration of 500 CFU (equivalent to a TTP of approximately 8.5 days) per 180µl of media for each co-culture well required.

*Note: If stock is highly concentrated, the stock should be diluted in several steps (e.g. serial 1:10 dilutions) to avoid pipetting very small volumes.*

*Note: If other mycobacterial species or strains are used, the optimum multiplicity of infection (MOI) for each strain should be determined prior. For an example MOI optimisation experimental design, please refer to Zelmer et al. ^32^.*

3.2.11 Add 180µl (containing 500 CFU) of the BCG final preparation to each sample well.

3.2.12 Incubate the 48-well plates in a CO _2_ incubator at 37°C for 96 hrs (4 days).

3.2.13 Supplement one MGIT tube with 800µl PANTA/enrichment to produce supplemented Middlebrook 7H9. Decant the contents into a fresh falcon tube for use in step 3.2.15.

3.2.14 Supplement 2 further MGIT tubes with 800µl PANTA/enrichment. These are the direct-to-MGIT inoculum controls.

3.2.15 Add an equal volume (180µl) of diluted BCG Pasteur prepared in step 3.2.10 to each of the 2 direct-to-MGIT controls. Using the extra supplemented Middlebrook 7H9 produced in step 3.2.13, make up the added volume to 500µl (so if 180µl of BCG preparation is added, add an additional 320µl of supplemented Middlebrook 7H9). Invert to mix, scan the barcode and place on the BACTEC MGIT machine. Refer to section 3.1.8 and section 3.1.10 for obtaining results.

### 3.3 MGIA day 4: Assay processing (summarised graphically in Figure 4)

**Figure 4.**
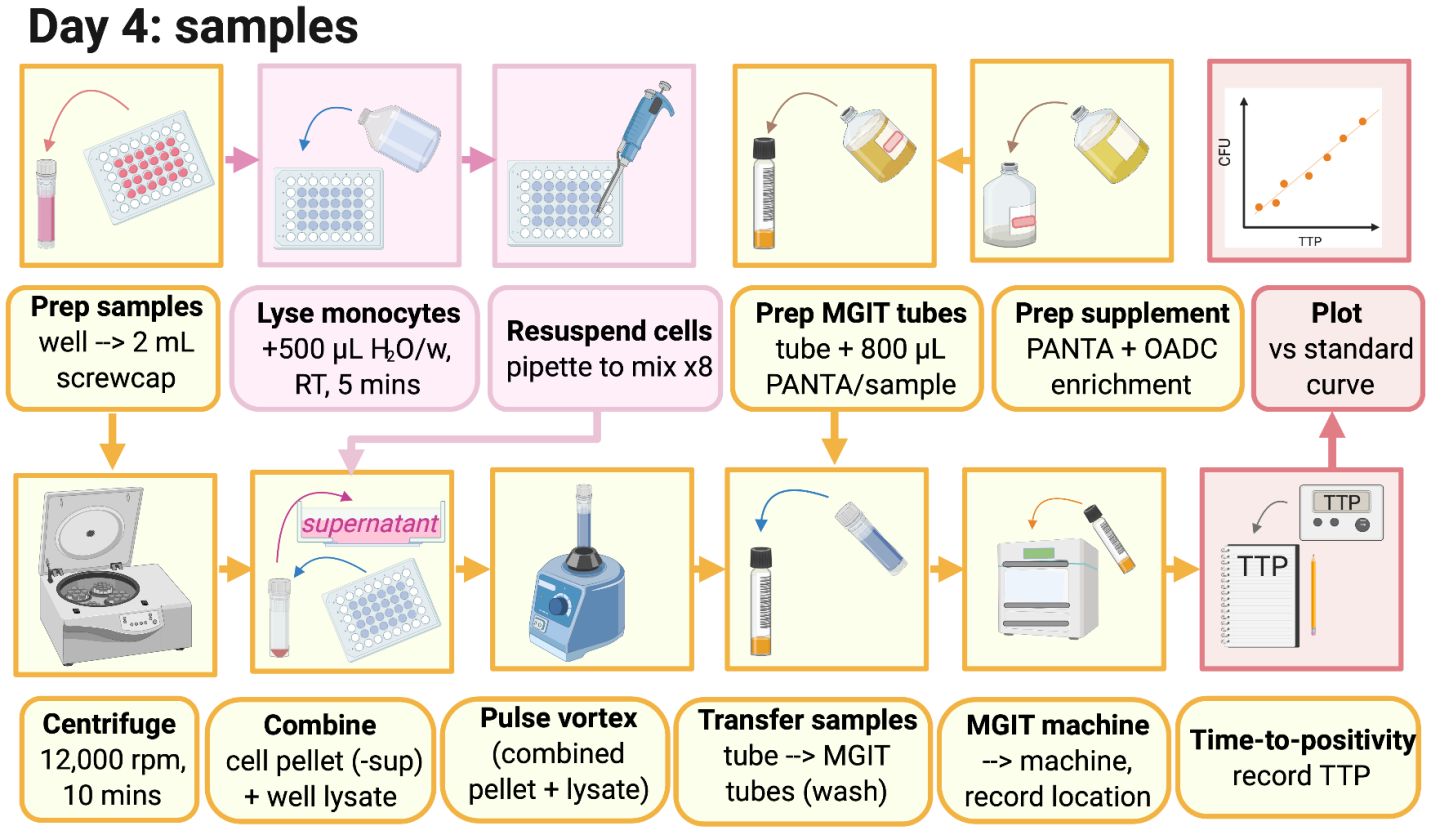
Day 4 MGIA processing (created with BioRender.com).

**Figure 5.**
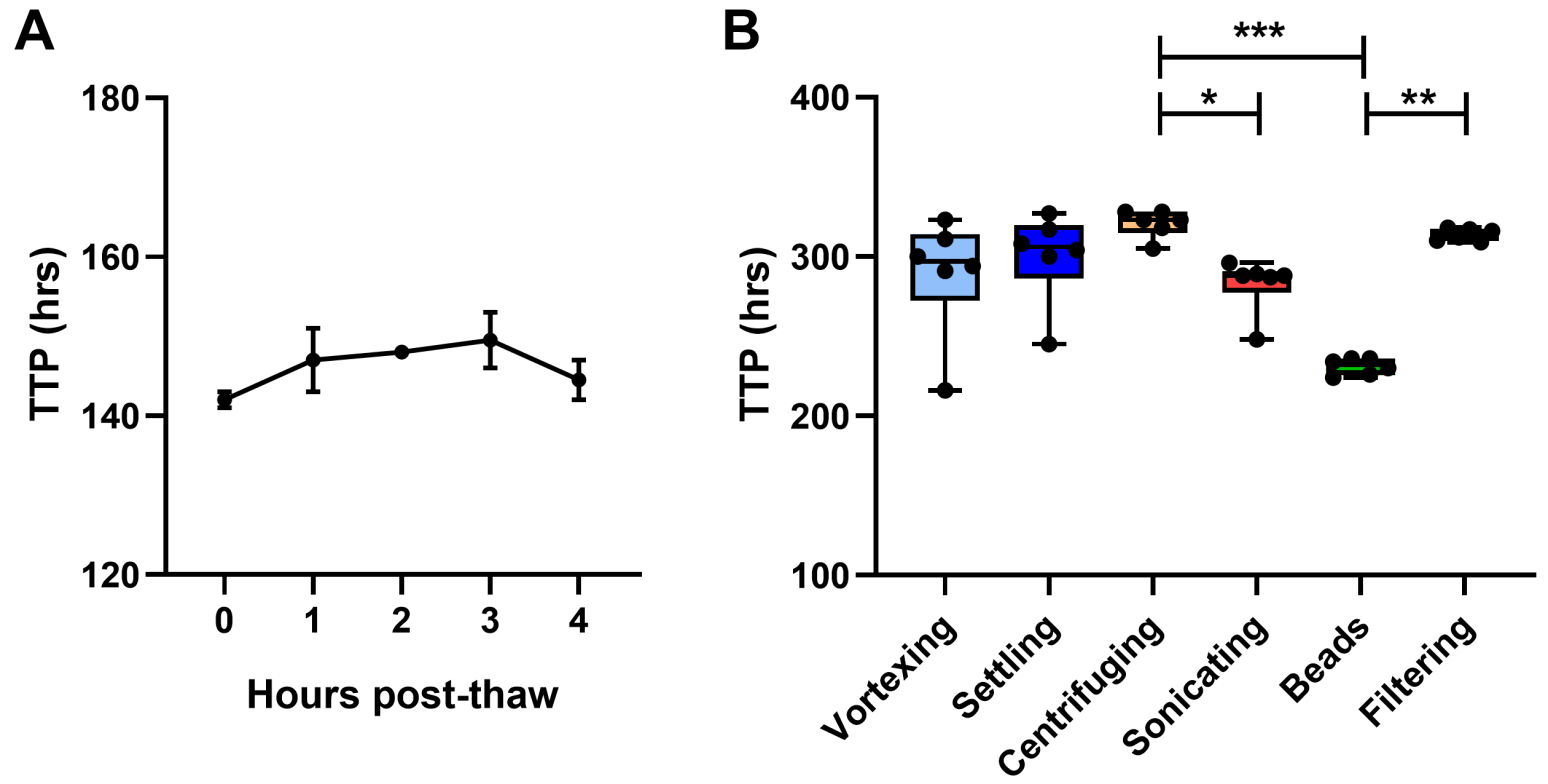
Mycobacterial stock preparation. The effect of **A**) time from thawing to inoculation and **B**) de-clumping method for mycobacterial stock on BACTEC MGIT time to positivity (TTP) were determined using in-tube co-cultures of human PBMC and BCG Pasteur. For **A**), points represent the mean of n=2 duplicate co-cultures with the standard error of the mean (SEM). For B), points represent n=6 individual replicate co-cultures, boxes indicate the median value with the interquartile range and whiskers indicate the minimum and maximum values. A Kruskal-Wallis test was performed with a Dunn’s multiple comparisons test, where * indicates a p-value of <0.05, ** indicates a p-value of <0.01, and *** indicates a p-value of <0.001.

**Figure 6.**
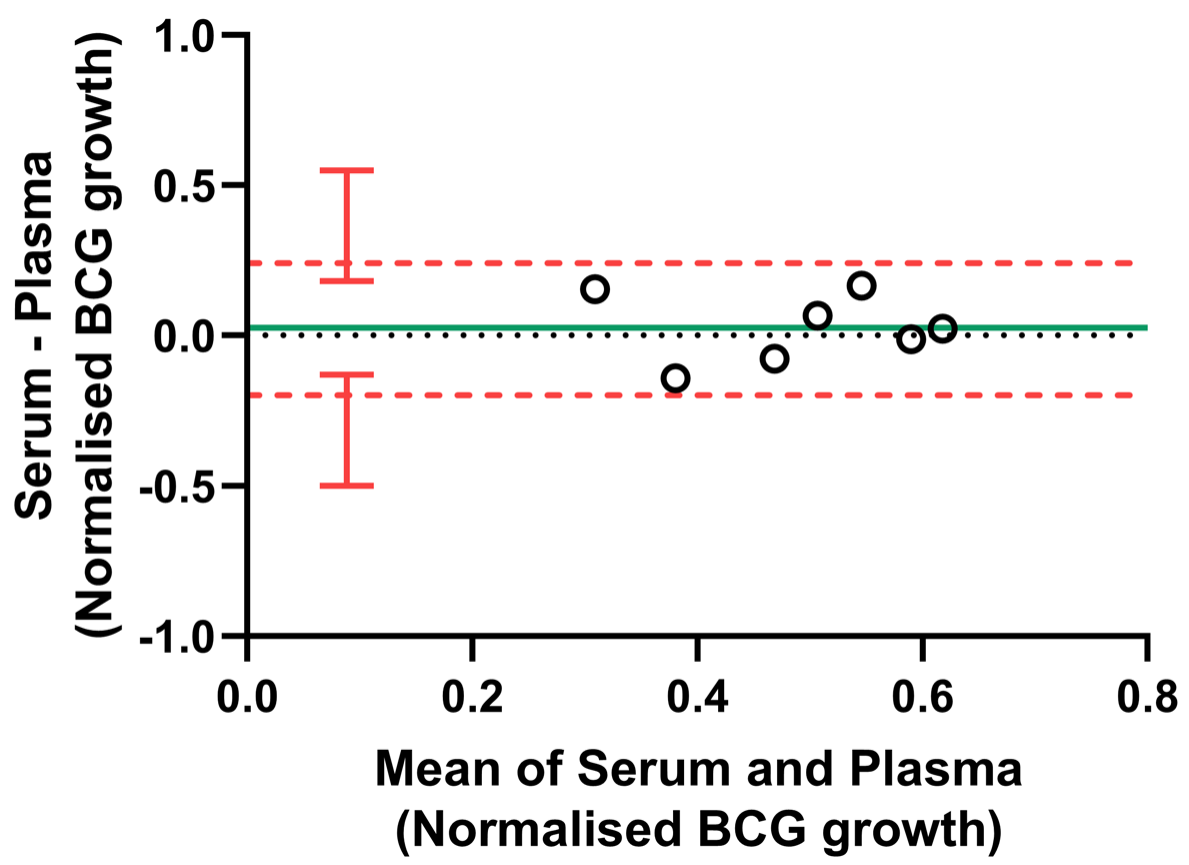
Bland-Altman plot comparing serum vs. plasma in the NHP direct MGIA. The NHP PBMC direct MGIA was conducted using either autologous serum or plasma for n=7 macaques. Co-cultures were performed in duplicate where sufficient numbers of cells were recovered. The solid green line indicates the mean difference between measurements and the dotted red line indicates the upper and lower limits of agreement (mean difference ± 1.96 standard deviation of the difference) with red vertical bars showing the 95% confidence intervals for the limits of agreement.

**Figure 7.**
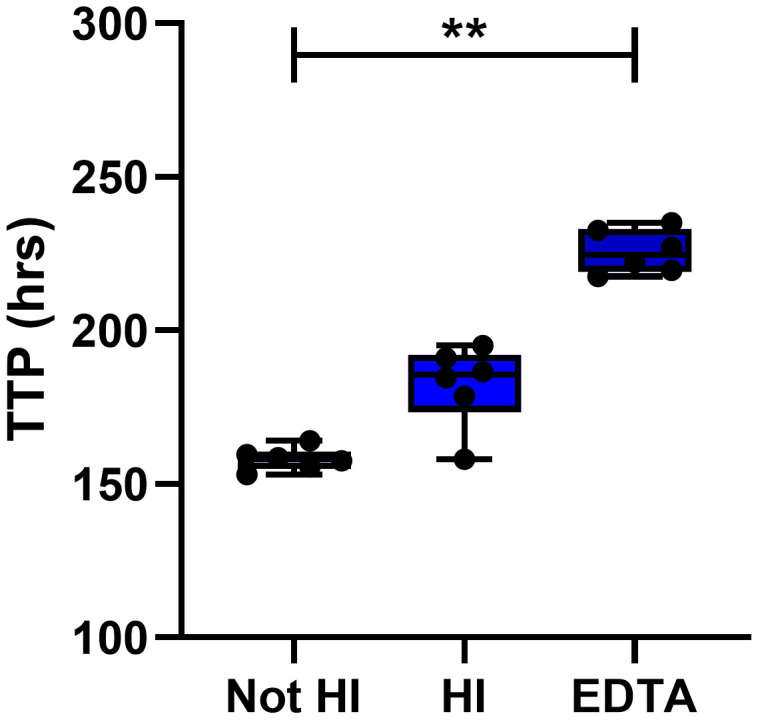
Effect of using heat inactivated serum or collecting blood in EDTA vacutainers. The effect of heat inactivating (HI) pooled human serum or of collecting blood in EDTA tubes was assessed using in-tube co-cultures for n=6 human PBMC. Points represent individuals with the mean of two replicate co-cultures, boxes indicate the median value with the interquartile range and whiskers indicate the minimum and maximum values. A Friedman test with Dunn’s correction for multiple comparisons was used where ** indicates a p-value of <0.01. TTP = MGIT time to positivity, HI = heat inactivated.

3.3.1 Before harvesting co-cultures, supplement 1 MGIT tube per culture well with 800μl PANTA enrichment and label.

3.3.2 Pipette the cultures in the well up and down three times, collect the liquid and transfer to a 2ml screw-cap tube.

3.3.3 Microcentrifuge tubes at 15,300 *g* for 10 minutes.

3.3.4 Add 500μl of sterile, tissue culture-grade water to each well, and incubate at room temperature for at least 5 minutes.

3.3.5 Remove 500μl of supernatant from the 2ml tubes, ensuring the pellet remains intact. Supernatant can be discarded unless required for later cytokine analysis.

*Note: Pellets appear as a small ‘smudge’ and are easily disturbed; particular care should be taken during this step to avoid disturbing the pellet.*

3.3.6 Pipette the water in the wells up and down ~8 times to detach monocytes that have attached to the bottom of the well (avoid forming bubbles as far as possible) and completely remove the water from the well, transferring it to the corresponding tube containing the cell/BCG pellet.

3.3.7 Pulse vortex for 1–2 seconds, and add all of the sample from the 2ml tube to the corresponding MGIT tube. Use some media from the MGIT tube to rinse the 2ml tube and add back to the same MGIT tube.

3.3.8 Invert all MGIT tubes to mix and place on the BACTEC MGIT instrument until positivity is reached (see section 3.1.8).

### 3.4 Data processing and reporting

3.4.1 Record TTP for control and sample MGIT tubes and convert to log _10_ CFU values using the corresponding stock standard curve generated in section 3.1.

*Note: Stored samples should be batched as far as possible; if more than one batch or experiment is to be directly compared, each sample read-out should be normalised to its corresponding direct-to-MGIT control (by subtracting the log _10_ CFU of the control from the log _10_ CFU of the sample) to account for differences in input inocula between assay runs.*

## 4.0 Results

### 4.1 Optimisation and characterisation studies

A range of optimisation and characterisation studies were conducted during the development of the direct MGIA. While some of these were performed using human cells (where specified) for reasons of ethics and sample availability, outcomes have informed the development of the macaque assay protocol.

***4.1.1 Mycobacterial stock***. In order to minimise the variability associated with low-titre mycobacterial inocula, two stock parameters were assessed: a) time from thawing to inoculation and b) de-clumping methods. Mycobacteria were thawed and added to duplicate human PBMC co-cultures every hour for 5 hours after resting on the bench at room temperature. Mycobacterial viability showed a progressive, albeit modest, decrease for the first 3 hours, before beginning to recover at 4 hours ( Figure 5A). Six methods of de-clumping were compared using 6 replicate in-tube co-cultures containing cells from the same human sample for each method: 1) vortexing for 5 minutes on the highest speed, 2) standing on the bench for 5 minutes to allow clumps to settle and then removing only the top fraction, 3) centrifuging at a low speed to bring clumps down and then removing only the top fraction, 4) sonicating for 2 minutes, 5) vortexing with 1mm borosilicate solid-glass beads for 2 minutes, and 6) syringing through a 5µM cellulose acetate filter.

Mycobacterial recovery was highest using the glass beads method, while other methods (particularly centrifuging and filtering) resulted in some loss of mycobacteria. BCG growth was significantly higher (lower TTP) following vortexing with glass beads compared with centrifuging or filtering (p=0.0002, Δ mean TTP = 90 hours; and p=0.008, Δ mean TTP = 83 hours respectively; Kruskal Wallis with Dunn’s multiple comparisons test, p=0.0002, Figure 5B). Reproducibility between replicates was greatest for glass beads and filtering (coefficient of variation, CV = 2.2% and 1.2% respectively), and poorest for vortexing (CV = 13%). Based on these findings, we recommend that mycobacterial stocks suffering from clumping should be vortexed with sterile 1mm borosilicate glass beads (Sigma Aldrich, UK) for 2 minutes prior to inoculation, and that inoculation should be conducted as soon after thawing as possible.

*M.tb* is the pathogen of interest and may be used as the mycobacterial inoculum in the direct NHP MGIA; indeed we have demonstrated a BCG-vaccine induced effect and a correlation with protection from *in vivo* mycobacterial challenge using whole blood from macaques co-cultured with *M.tb* H37Rv ^37^. However, a similar MGIA kinetic was observed whether BCG or *M.tb* was used as the inoculum, with a correlation between the two measures ^37^. Such an association has also been reported in the human direct MGIA ^28, 44^. In the NHP direct MGIA, we observed improved intra-assay reproducibility using *M.tb* compared with BCG which may have improved ability to detect a correlation with *in vivo* protection. However, using BCG increased sensitivity to observe a vaccine response (post-vaccination growth – baseline growth), and it was this measure that correlated most consistently with *in vivo* protection in our studies ^37^. On balance, we chose to pursue assay development using BCG to aid transferability by negating the need for high containment level laboratory facilities.

***4.1.2 Co-culture conditions***

**4.1.2.1 Whole blood vs. PBMC**

While whole blood may represent the most *ex vivo* sample, we previously reported a correlation between mean corpuscular haemoglobin (Hb) and mycobacterial growth in the human direct MGIA ^35^. Furthermore, addition of either Hb or ferric ammonium citrate to both human and macaque PBMC MGIA co-cultures enhanced mycobacterial growth, whereas the addition of the iron chelator deferoxamine reduced it ^35^. Taken together, these data indicate an association between Hb/iron and mycobacterial growth, likely via the heme iron uptake pathway ^45^. This effect is particularly pertinent in preclinical models such as the macaque, where blood collections can perturb Hb levels. Indeed, while levels remained within the normal range for the species, we observed a significant decrease in Hb concentration at 4 and 8 weeks relative to baseline following a fortnightly blood collection regimen in rhesus macaques ^35^. Thus, while the direct whole blood MGIA may be appropriate to studies that use infrequent longitudinal sample collections or where variation in clinical parameters including Hb levels form part of the overall response, it may confound measures of vaccine-induced control of mycobacterial growth and reduce sensitivity to detect a vaccine response.

In humans, the direct PBMC MGIA demonstrated a stronger primary vaccine effect and greater reproducibility over repeated baseline bleeds compared with whole blood, most likely due to the evaluation of longitudinal PBMC samples in one batch ^30^. Ability to batch samples in this way also improves logistical feasibility and transferability, particularly to institutes without immediate access to a BACTEC MGIT instrument. Furthermore, the use of cryopreserved cells enables additional retrospective studies of samples from historical NHP vaccine studies for validation and exploratory work, thus reducing the number of animals used. We therefore focussed our assay development efforts on the PBMC compartment. Importantly, we previously reported a significant influence of penicillin-streptomycin (P/S) antibiotics on mycobacterial growth if included in the culture medium during thawing and the subsequent cell rest period for human cells ^30^. Optimisation experiments using 1×10 ^6^ cells from n=3 volunteers in duplicate and a BCG inoculum of ~800 CFU in the in-tube MGIA confirmed a pronounced inhibitory effect of P/S if present in the culture medium post-thawing but not pre-freezing (mean TTP with P/S pre-freezing and post-thawing = 262 hours, STDEV = 26; P/S post-freezing only = 281 hours, STDEV = 7.4; P/S pre-freezing only = 113 hours, STDEV = 4; no P/S = 109 hours, STDEV = 3.6). Although penicillin has no reported activity against mycobacteria, streptomycin is a broad-spectrum bactericidal drug used as a first-line treatment for TB ^46^. Uptake of streptomycin into human cells does occur, where it is sequestered in lysosomes and redistributed into the cytosol and concentrated ^47^. Therefore it is likely that, despite washing cells following the cell rest, residual or retained streptomycin remained present in the co-culture. We therefore recommend the use of P/S in the pre-freezing medium only.

**4.1.2.2 Multiplicity of infection**

We previously demonstrated that reducing the MOI by increasing cell number rather than reducing mycobacterial inoculum increases ability to detect a BCG vaccine induced response using the NHP direct MGIA ^37^. This was consistent with findings using both the mouse and human direct MGIAs ^29, 32^. We also showed that repeatability and ability to detect a vaccine induced response is improved by co-culturing in static 48-well plates compared with rotating screw-cap tubes ^37^, again reflecting findings in other species ^29, 34^. Based on these observations, the limitations of cell availability, and to ensure consistency with the equivalent human assay ^29^, we recommend the conditions of 3 × 10 ^6^ cells co-cultured in 48-well plates with 500 CFU BCG as described in section 3.0. However, an alternative protocol using 1 × 10 ^6^ cells in sealed, rotating 2ml screw-cap tubes has been successfully applied in humans, and used in the NHP model to demonstrate improved control of mycobacterial growth following *M.tb* infection ^29, 36^. Some researchers consider that the in-tube protocol may be applied where cell number is limiting and biological effects strong, and can be used to further dissect the mechanism of mycobacterial growth control ^29, 48^. Details of this alternative method and the associated protocol may be found in the report of optimisation and standardisation of the human direct MGIA ^29^.

**4.1.2.3 Serum**

We recommend the addition of autologous time-point matched serum to co-cultures to resemble *ex vivo* conditions as closely as possible and ensure that any effects of vaccination mediated by serum factors are taken into account. We recently demonstrated that the addition of autologous serum contributes to improved control of mycobacterial growth following BCG vaccination in the human direct PBMC MGIA [Bitencourt *et al*. submitted ^49^]. Using autologous serum also has the 3Rs benefit of not using foetal bovine serum (FBS) which has ethical implications ^50^. We titrated the serum concentration using in-tube human PBMC co-cultures (n=4), and found that mycobacterial growth was similar when adding 5, 10 or 20% serum (mean TTP = 285, 257 and 316 hours respectively), but increased when serum was at a concentration of 30% (TTP = 180 hours). While 5–20% is a standard serum concentration for cell culture, 30% may be detrimental to cell viability, allowing mycobacteria to proliferate unchecked.

Due to limitations regarding the maximum blood volume permitted for collection from macaques, plasma may be a more feasible alternative to serum. As specific antibodies are likely the main component of serum contributing to control of mycobacterial growth in the MGIA, we compared levels of PPD-specific IgM, IgG and IgA between serum and plasma from matched animals at baseline. In all cases there was a strong correlation, although serum contained modestly but significantly higher levels of specific antibodies at most time-points measured [Bitencourt *et al.* submitted ^49^]. We therefore compared the use of autologous serum vs. autologous plasma in the direct NHP MGIA co-culture (n=7 animals), in which other components such as complement factors may also contribute to functional control of mycobacterial growth, and observed an intraclass correlation coefficient (ICC) of 0.58 (moderate agreement). As shown by Bland-Altman analysis relating the difference between paired measurements to the mean of the pair, there was minimal bias between the two methods (mean bias = 0.025). Furthermore, all samples were within the 95% limits of agreement (the interval of 1.96 standard deviations of the measurement differences either side of the mean difference), which extended from -0.20 (95% CI, -0.50 to -0.13) to 0.25 (95% CI, 0.18 to 0.55) log _10_ CFU ( Figure 6). Although the sample size was small and there is some in inherent intra-assay variability, this suggests that plasma may be substituted where serum is unavailable or limited in volume, but we do not recommend using the two samples interchangeably within a single experiment or direct comparison.

The effect of heat inactivating serum was assessed by measuring mycobacterial growth at the end of in-tube n=6 human PBMC co-cultures. Mycobacterial growth was lower when co-cultures contained serum that had been heat-inactivated compared with serum that had not been heat inactivated, but this was not statistically significant by Friedman with Dunn’s correction for multiple comparisons (Δ mean TTP = 24 hours; Friedman with Dunn’s correction for multiple comparisons, Figure 7). It has been reported that heat inactivation of serum decreases uptake of mycobacteria into monocytes due to the destruction of complement ^51^. As monocytes provide the target host cell for mycobacterial survival and replication, a decrease in monocyte invasion may lead to decreased mycobacterial growth. Finally, we compared serum/plasma separated from blood collected in either serum clot-activator or Ethylenediaminetetraacetic acid (EDTA) vacutainers. Adding plasma separated from an EDTA vacutainer to the MGIA co-culture resulted in significant inhibition of mycobacterial growth (p=0.003, Δ mean TTP = 68 hours; Friedman with Dunn’s correction for multiple comparisons, Figure 7). EDTA has been shown to have anti-tubercular activity and has even been suggested for potential use in treatment of drug-resistant TB ^52^. Based on these findings, we recommend that autologous serum/plasma should be added to a final concentration of 20%, should not be heat-inactivated and should not be collected in vacutainers containing EDTA.

***4.1.3 Day 4 processing***. At the end of the 96-hour co-culture period, cells are lysed to release intracellular mycobacteria. We previously compared mycobacterial recovery under 5 different cell lysis conditions using the human in-tube direct PBMC MGIA: 1) none, 2) sterile water, 3) PBS with Tween 20, 4) 0.2% Saponin, and 5) 0.067% Sodium Dodecyl (lauryl) Sulfate (SDS) across three different sites. BCG recovery was comparable across conditions at all sites ^29^. While the cell lysis step can thus be omitted for the in-tube protocol, it must be included in the recommended 48-well plate protocol to ensure that mycobacteria are released from monocytes that have adhered to the well surface; we suggest the use of sterile water to maximise transferability.

***4.1.4 Characterisation of intra- and inter-assay reproducibility***. We previously characterised the repeatability of the direct NHP MGIA at 3 different sites. The median CV between replicate co-cultures was 2.69% (range 0.59 to 6.12%, n=8), 1.67% (range 0.78 to 8.52%, n=5) and 2.71% (range 0 to 7.33%, n=5) at sites 1, 2 and 3 respectively. The ICC values were 0.90 (‘almost perfect’ agreement), 0.34 (‘fair’ agreement) and 0.95 (‘almost perfect’ agreement) respectively ^37^. A single sample set (n=8) was assayed on two separate occasions at the same site to assess inter-assay precision. The median CV between assay runs was 6.83% (range 2.13 to 7.76%) with an ICC value of 0.80 (‘substantial’ agreement). While there was a strong consistency agreement, mycobacterial growth was systematically higher (indicated by a shorter TTP) in run 2. The most likely cause is a difference in inoculum due to differences in titre or viability between mycobacteria stock vials. However, as shown by Bland-Altman analysis, the bias was not fully compensated by normalising growth using the direct-to-MGIT control (mean bias = 0.39). We thus recommend assaying all samples from different treatment groups or across a longitudinal time-course in a single batch. It should be noted that all samples between the two runs were within the 95% limits of agreement, which extended from 0.12 (95% CI, -0.19 to 0.21) to 0.66 (95% CI, 0.58 to 0.97) log _10_ CFU ^37^, but further work is required to achieve absolute agreement.

### 4.2 Validation studies

The biological relevance of the MGIA as a surrogate measure of vaccine efficacy can only be confirmed by comparing outcomes with levels of protection following *in vivo* mycobacterial challenge or infection. Similar assessments have been conducted of the malaria growth inhibition assay in relation to protection from controlled malaria infection in NHPs and humans ^15, 53– 56^. This has previously been achieved for the human and murine direct MGIAs at the group level ^22, 31, 33, 57^. However, validation at an individual level would be more stringent given the variability in BCG-induced protection between individuals and animals ^58, 59^. We recently described an association between mycobacterial growth in the direct PBMC MGIA and outcome of *in vivo* intradermal BCG infection at the individual level in humans ^33^. BCG was used in this study as a potential surrogate challenge agent for virulent *M.tb*, which cannot ethically be used in human infection studies ^60^. The NHP model provides an opportunity to validate the assay against direct measures of protection from *M.tb* as well as BCG infection, allowing greater confidence in the relevance of the human assay such that preclinical models may ultimately be replaced in some settings.

As previously reported, we used samples from BCG vaccinated NHPs across four different studies to evaluate biological validity of the NHP MGIA ^37^. In the first study, there was a significant correlation between *M.tb* growth in the whole blood MGIA at the peak of response and the number of BCG CFU recovered from the axillary lymph nodes following *in vivo* BCG challenge. There was a more pronounced association between MGIA vaccine response (post-vaccination growth – baseline growth) and lymph node CFU. MGIA vaccine response at the peak time-point also correlated with multiple measures of protection following *in vivo M.tb* challenge in a further two studies ^37^. This suggests that the magnitude of vaccine response relative to baseline (which is akin to fold change and captures more information in a single measure) is a more representative measure of *in vivo* protection than absolute inhibition at a given time-point. This correlation between MGIA outcome and measures of protection from *in vivo* challenge with either BCG or *M.tb* at an individual animal level affords confidence that the assay is measuring a biologically meaningful response, although further validation is required alongside ongoing *in vivo* studies.

## 5.0 Discussion

### 5.1 Transferability

One of the objectives when developing the direct MGIA was to provide an assay that was, technically and logistically, as simple as possible to maximise reproducibility and transferability ^28^. In the absence of a validated correlate of protection, we also chose not to include stimulation or expansion steps to avoid biasing, or over-representing certain aspects of, the immune response. We previously sought to transfer and harmonise the protocol defined here to ensure that the 3Rs impact is maximised and that comparable information can be extracted from ongoing and future studies of different preclinical vaccine candidates across organisations. As recommended by Smith *et al*., we conducted side-by-side operator training at end-user institutes ^61^, and then assessed reproducibility (variation between multiple determinations of a single sample analysed at different laboratories or sites ^62^) by conducting inter-site comparisons between sites 1 and 2 and sites 1 and 3 using two shared sample sets. Between sites 1 and 2, the median CV was 14.19% (range 11.57 to 17.29%, n=7) with an intraclass correlation coefficient (ICC) value of 0.57 (‘moderate’ agreement). Between sites 1 and 3, the median CV was 3.17% (range 0.39 to 8.62%, n=8) with an ICC of 0.83 (‘almost perfect’ agreement) ^37^. The comparison between sites 1 and 2 resulted in lower inter-site reproducibility, which may have been due to the more homogeneous sample set used which had similar levels of growth control across animals. We therefore selected a sample set with a broader dynamic range for the comparison between sites 1 and 3, and observed a close mirroring in the pattern of control ^37^.

Our reproducibility values were comparable to those reported for the human PBMC MGIA ^29^ and were well within the 50% limit of acceptable variation suggested by Tuomela *et al*. for the measurement of a bacterial target in a cell-based assay ^62^. However, we did observe a systematic difference in the site 1–2 comparison. Again, normalising growth values using the corresponding direct-to-MGIT control did not fully compensate for this bias and further work is required to achieve absolute agreement ^37^. However, all samples were within the 95% limits of agreement, which extended from -0.61 (95% CI, -0.89 to -0.54) to -0.21 (95% CI, -0.27 to 0.07) log _10_ CFU for the site 1–2 comparison and -0.26 (95% CI, -0.70 to -0.15) to 0.49 (95% CI, 0.37 to 0.93) log _10_ CFU for the site 1–3 comparison. Importantly the delta between the highest and lowest values was consistent between sites, and given that the magnitude of vaccine response (post-vaccination growth – baseline growth) appears to be the most relevant measure as a surrogate of protective efficacy, systematic differences may be less problematic ^37^. The delta between baseline and post-vaccination time-points, or between vaccinated and unvaccinated animals, should thus be considered in comparisons of vaccine efficacy measured at different sites rather than absolute growth values.

The main barrier to uptake of this assay by other potential end-users is the requirement for a BACTEC MGIT machine and the cost of associated reagents. While we recommend this quantification system as a faster, simpler, more sensitive and more objective alternative to CFU plating on solid agar, Kolibab *et al*. have demonstrated a highly significant linear inverse correlation between BACTEC MGIT TTP and CFU on solid agar following a 7 day MGIA using mouse splenocyte co-cultured with bone marrow macrophages ^63^. It may therefore be possible to use traditional colony counting in resource-limited settings. That said, the BACTEC MGIT machine is a widely-used TB diagnostic tool available in most hospitals worldwide and many academic medical research groups have indirect access. Furthermore, using cryopreserved PBMC permits the batching of samples which improves logistical feasibility for those with limited MGIT access compared with whole blood assays, which must be run in real-time at multiple time-points. An additional potential barrier to uptake of MGIAs is access to high containment level facilities for the handling of virulent *M.tb*. For this reason, we focussed our optimisation work around the use of BCG as a surrogate agent of *in vitro* infection as discussed in section 4.1.1.

### 5.2 Translatability

The relative simplicity of the direct MGIA method described makes it highly translatable across host species and compartments ^28^. We have demonstrated optimisation and application of the assay using splenocytes ^31, 32, 43^ and, more recently, lung cells ^64^ from mice. Applying the assay in place of *M.tb* challenge experiments locally has downgraded the severity of many of our murine TB vaccine studies from ‘Moderate’ to ‘Mild’ as vaccination is the only *in vivo* procedure required. Other groups have also reported use of the murine assay ^34, 57^. Attempts to adapt the assay for use in the bovine model have, however, been less successful ^65^, suggesting that translation may not be straightforward in all cases. In humans, we have optimised and harmonised the direct MGIA as part of the FP7 European Research Infrastructures for Poverty Related Diseases (EURIPRED) consortium ^29^, applied it to demonstrate a BCG vaccine effect ^30^, and validated it against protection from *in vivo* experimental BCG infection ^33^. Studies by our group and others demonstrate how the direct MGIA may be employed to address different aspects of TB research including clinical studies of TB patients ^44, 48, 66, 67^, coinfections ^68^, and underlying immune mechanisms of protection ^48, 69– 73^. Indeed, the direct NHP MGIA has also been applied to demonstrate improved control of mycobacterial growth following *M.tb* infection ^36^, consistent with findings in recently *M.tb*-infected humans ^44, 48^. Beyond TB, we have recently adapted the assay for use with other pathogens including *S. aureus* and *K. pneumoniae* to explore the potential non-specific effects of BCG vaccination in humans [Wilkie M and Tanner R, unpublished data]. 

### 5.3 Measures of success/acceptance

Based on our experience of standardisation and harmonisation of the NHP MGIA, we recommend that repeatability between replicate co-cultures and precision between different runs of the same samples should be below 10% CV and above 0.5 ICC. Ideally an inter-site comparison between the developer and end-user site should be conducted using a shared sample set, with a reproducibility cut-off of below 15% CV and above 0.5 ICC. Bland-Altman analyses for both inter-assay and inter-site comparisons allowed us to define limits of agreement (as reported in section 4.1.5 and section 5.1), which may be considered estimates of population parameters, although it should be noted that the systematic biases described will influence these values. Comparisons of standard curves from a common stock between sites would also aid confidence in initial assay transfer. The ultimate test of acceptance is conducting the NHP MGIA alongside one or more *in vivo* mycobacterial infection stud(ies) and demonstrating a significant association between outcomes. As BCG is currently the only licenced TB vaccine, ability to detect a BCG vaccine-induced response is the benchmark for assessing correlates of protection, and could be used in this context (using samples where BCG is known to have conferred protection *in vivo*). A more stringent measure would be correlating MGIA outcomes with measures of *in vivo* protection mediated by BCG and other TB vaccine candidates at an individual animal level, as we have previously described for BCG vaccination ^37^.

### 5.4 Scientific and 3Rs benefits and impact

***5.4.1 Scientific benefits***. Broadly speaking, a reliable and validated MGIA for use with samples from immunised NHPs would permit high-throughput cost-effective evaluation of vaccine candidates, and down-selection of those going forward into *in vivo* efficacy testing; this would ultimately expedite the development of a much-needed effective TB vaccine. The direct MGIA also provides a tractable system for the assessment of immune mechanisms underlying the control of mycobacterial growth; manipulation of immune parameters in this way (e.g. cell depletions) is often not logistically or ethically feasible *in vivo*. Findings may further inform our understanding of protective immunity from TB and thus direct improved vaccine design as well as development of diagnostic and therapeutic tools. The NHP MGIA in particular offers the opportunity to biologically validate the assay through correlation with direct measures of protection from *in vivo M.tb* challenge on an individual animal basis. This is not possible using mice (where animals must be euthanised for the splenocyte MGIA and can therefore only be correlated by group) or humans (where *M.tb* challenge is not ethically viable). Such validation allows bridging to use in target species including humans where direct measures of protection cannot be obtained.

***5.4.2 3Rs benefits***. The process of early testing of TB vaccine candidates in NHP models could be refined by using the MGIA in place of *in vivo* infection with pathogenic *M.tb*. Furthermore, the number of NHPs used in TB vaccine testing and associated immunology studies could be reduced, as the MGIA allows:

a) Testing of multiple conditions (for example different mycobacterial clinical isolates and immunological mechanisms) using cells from a single group, rather than requiring multiple groups of animals.
b) Down-selection of vaccine candidates at an early stage of development such that fewer go forward to *in vivo* efficacy testing.

Ultimately, biological validation in NHPs allows bridging of the assay to use in target species including humans which may replace the use of preclinical models in some settings.

## Data availability

### Underlying data

Figshare: NHP MGIA methods optimisation experiments, https://doi.org/10.6084/m9.figshare.14040074.v2 ^74^.

### Reporting guidelines

Figshare: ARRIVE checklist for ‘The in vitro direct mycobacterial growth inhibition assay (MGIA) for the early evaluation of TB vaccine candidates and assessment of protective immunity: a protocol for non-human primate cells’, https://doi.org/10.6084/m9.figshare.14040074.v2 ^74^.

Data are available under the terms of the Creative Commons Attribution 4.0 International license (CC-BY 4.0).
