# Supplementary material for: The in vitro direct mycobacterial growth inhibition assay (MGIA) for the early evaluation of TB vaccine candidates and assessment of protective immunity: a protocol for non-human primate cells
Source: F1000Res. 2021 Sep 23;10:257. Originally published 2021 Mar 30. [Version 2] doi: 10.12688/f1000research.51640.2 (PMC8097740; doi:10.12688/f1000research.51640.2)
Supplement: Peer review history

# Peer review: Reviewer response for version 1

**DOI:** 10.5256/f1000research.54829.r82511

**Published:** 2021-05-04

**Authors:** Delia Goletti

**Affiliations:** 1 Translational Research Unit, "L. Spallanzani" National Institute for Infectious Diseases (INMI), Rome, Italy

'The *in vitro *direct mycobacterial growth inhibition assay (MGIA) for the early evaluation of TB vaccine candidates and assessment of protective immunity: a protocol for non-human primate cells' by Tanner *et al. *describes the method for the use of a direct MGIA assay using NHP cells and

 autologous serum acquired from vaccination studies. The manuscript is well written and the figures are very clear.  

 The authors emphasize the use of the reported tests to reduce the number of animal models used in the TB vaccine research pipeline as well as negating the use of virulent *M.tb *challenge (thereby improving lifetime welfare of the animal) with the added benefit of investigating the intricacies of immune cell interactions in post-vaccination responses.

 The methodology section is very accurate in a way that the reader may easily perform the test. The authors add key points within the method to enhance the reproducibility.

 The manuscript is complete.

Is the rationale for developing the new method (or application) clearly explained?

Yes

Is the description of the method technically sound?

Yes

Are the conclusions about the method and its performance adequately supported by the findings presented in the article?

Yes

If any results are presented, are all the source data underlying the results available to ensure full reproducibility?

Yes

Are sufficient details provided to allow replication of the method development and its use by others?

Yes

Reviewer Expertise:

Infectious diseases specialist. Scientist studing the immune response to pathogens.

I confirm that I have read this submission and believe that I have an appropriate level of expertise to confirm that it is of an acceptable scientific standard.

# Peer review: Reviewer response for version 1

**DOI:** 10.5256/f1000research.54829.r82506

**Published:** 2021-04-15

**Authors:** Amanda J. Gibson

**Affiliations:** 1 IBERS, Aberystwyth Universiy, Aberystwyth, UK

The *in vitro* direct mycobacterial growth inhibition assay (MGIA) for the early evaluation of TB vaccine candidates and assessment of protective immunity: a protocol for non-human primate cells by Tanner *et al.* describes the method for the use of a direct MGIA assay using NHP cells and autologous serum acquired from vaccination studies. Particular highlights are the use of the described assays to reduce the number of NHPs used in the TB vaccine research pipeline as well as negating the use of virulent *M.tb *challenge (thereby improving lifetime welfare of NHPs) with the added benefit of investigating the intricacies of immune cell interactions in post-vaccination responses. Method is well described, both in preceding rationale and in a technical stepwise fashion such that the reader may easily replicate. To enhance transferability of the method, the authors suggest alternative components to support completion of the method in more limited laboratory settings. Key points within the method that enhance reproducibility are also highlighted with extensive notes to assist the reader in reproducing the method with repeatable results.

 While the method is well described and clear such that replication by others would be possible, there are some minor improvements (listed below) that may be considered to further support the reader in adopting the MGIA within another laboratory environment.

- Table 2 – indicate glucose supplementation level of RPMI 1640 formulation and ensure formulation number is correct for both RPMI entries.
- Section 2.4 – take care to include spaces between numbers and units e.g. 2mM should read 2 mM throughout the method sections, in addition please take care to remain consistent with time notation; use of min, minutes, hr, hours, h are used interchangeably throughout the methods sections and graphical representations. Should addition of sodium pyruvate be included here as in Section 3.2?
- Section 3.1.2 – specifies sterile PBS, but Figure 2 describes PBS-Tw80.
- Section 3.1.9 – this describes using Miles and Misra spotting for the determination of CFU – Table 3 specifies the use of 60 mm petri dishes, is this correct? 3 x 20 ul spots on 60 mm plates is technically challenging without pre-drying of agar plates to ensure robust surface tension of dilution spots to prevent spreading. Guiding the reader to an appropriate reference of this CFU technique may assist those not familiar with Miles and Misra spotting for CFU counting.
- Figure 2 – please describe the significance or meaning of the red asterisk after BCG.
- Section 3.2.2 – please specify the concentrations used for L-glutamine and sodium pyruvate.
- Section 3.2. 7 – 25mM HEPES in underlined.
- Figure 3 – ensure centrifuge speeds are consistent with manuscript text, define R10 media.
- Section 3.2. 10 – PEN/STREP is capitalised.
- Section 3.2. 15 – should the added volume be made to 600 ul not 500 ul to remain consistent with other controls and samples?
- Figure 4 – ensure centrifuge speed notation is consistent with manuscript text.
- Section 4.0 Results – second sentence use of “outcomes” is not clear.
- Section 4.1.2.1 – is there a word missing between most and ex vivo in the first sentence e.g. used?

Is the rationale for developing the new method (or application) clearly explained?

Yes

Is the description of the method technically sound?

Yes

Are the conclusions about the method and its performance adequately supported by the findings presented in the article?

Yes

If any results are presented, are all the source data underlying the results available to ensure full reproducibility?

Yes

Are sufficient details provided to allow replication of the method development and its use by others?

Yes

Reviewer Expertise:

Innate Immunology, Mycobacteriology

I confirm that I have read this submission and believe that I have an appropriate level of expertise to confirm that it is of an acceptable scientific standard.

# Response

**Published:** 2021-09-15

**Authors:** Rachel Tanner

**Affiliations:** University of Oxford, UK

Many thanks for the very helpful comments, which have resulted in a corrected and improved version of the paper.

**Table 2 – indicate glucose supplementation level of RPMI 1640 formulation and ensure formulation number is correct for both RPMI entries.**

 The glucose supplementation level of RPMI-1640 (1.8-2.2 g/l) has now been added and the formulation number has been corrected to RPMI-1640 for both entries.

**Section 2.4 – take care to include spaces between numbers and units e.g. 2mM should read 2 mM throughout the method sections, in addition please take care to remain consistent with time notation; use of min, minutes, hr, hours, h are used interchangeably throughout the methods sections and graphical representations. Should addition of sodium pyruvate be included here as in Section 3.2?**

 Spaces between numbers and units have now been added and time notation made consistently longhand throughout the text and figures. Sodium pyruvate and its concentration has now been added where relevant.

**Section 3.1.2 – specifies sterile PBS, but Figure 2 describes PBS-Tw80.**

 Tween 80 has been removed from Figure 2 and the reagents list, as this should read sterile PBS only.

**Section 3.1.9 – this describes using Miles and Misra spotting for the determination of CFU – Table 3 specifies the use of 60 mm petri dishes, is this correct? 3 x 20 ul spots on 60 mm plates is technically challenging without pre-drying of agar plates to ensure robust surface tension of dilution spots to prevent spreading. Guiding the reader to an appropriate reference of this CFU technique may assist those not familiar with Miles and Misra spotting for CFU counting.**

 Table 3 has been corrected to specify 100mm petri dishes, and more information on the plating method including a reference to Miles and Misra spotting has been added to section 3.1.9.

**Figure 2 – please describe the significance or meaning of the red asterisk after BCG.**

 This asterisk has been removed to prevent confusion.

**Section 3.2.2 – please specify the concentrations used for L-glutamine and sodium pyruvate.**

 The concentration for L-glutamine (2 mM) and sodium pyruvate (1 mM) have now been added.

**Section 3.2. 7 – 25mM HEPES in underlined.**

 This has been corrected to remove the underlining.

**Figure 3 – ensure centrifuge speeds are consistent with manuscript text, define R10 media.**

 These have been amended to ensure consistency between figures and text in the unit of speed (g), and the reference to R10 in the figure has been removed (it now reads ‘Media + HEPES’).

**Section 3.2. 10 – PEN/STREP is capitalised.**

 All cases of capitalised PEN/STREP have been amended to lower-case.

**Section 3.2. 15 – should the added volume be made to 600 ul not 500 ul to remain consistent with other controls and samples?**

 This has now been amended accordingly.

**Figure 4 – ensure centrifuge speed notation is consistent with manuscript text.**

 This has been amended accordingly to ensure all speeds are in ‘g’.

**Section 4.0 Results – second sentence use of “outcomes” is not clear.**

 This has been changed to ‘results’ for clarity.

**Section 4.1.2.1 – is there a word missing between most and ex vivo in the first sentence e.g. used?**

 This has been reworded for clarity.
